# Prostaglandin E_2_ Reduces the Release and Infectivity of New Cell-Free Virions and Cell-To-Cell HIV-1 Transfer

**DOI:** 10.1371/journal.pone.0085230

**Published:** 2014-02-25

**Authors:** María Isabel Clemente, Susana Álvarez, María Jesús Serramía, Marta Martínez-Bonet, María Ángeles Muñoz-Fernández

**Affiliations:** 1 Laboratorio InmunoBiología Molecular, Hospital General Universitario Gregorio Marañón, Madrid, Spain; 2 Instituto de Investigación Sanitaria Gregorio Marañón, Madrid, Spain; 3 Networking Research Center on Bioengineering, Biomaterials and Nanomedicine (CIBER-BBN), Zaragoza, Spain; University of Missouri-Kansas City, United States of America

## Abstract

**Background:**

The course of human immunodeficiency virus type-1 (HIV-1) infection is influenced by a complex interplay between viral and host factors. HIV infection stimulates several proinflammatory genes, such as cyclooxigense-2 (COX-2), which leads to an increase in prostaglandin (PG) levels in the plasma of HIV-1-infected patients. These genes play an indeterminate role in HIV replication and pathogenesis. The effect of prostaglandin E_2_ (PGE_2_) on HIV infection is quite controversial and even contradictory, so we sought to determine the role of PGE_2_ and the signal transduction pathways involved in HIV infection to elucidate possible new targets for antiretrovirals.

**Results:**

Our results suggest that PGE_2_ post-infection treatment acts in the late stages of the viral cycle to reduce HIV replication. Interestingly, viral protein synthesis was not affected, but a loss of progeny virus production was observed. No modulation of CD4 CXCR4 and CCR5 receptor expression, cell proliferation, or activation after PGE_2_ treatment was detected. Moreover, PGE_2_ induced an increase in intracellular cAMP (cyclic AMP) levels through the EP2/EP4 receptors. PGE_2_ effects were mimicked by dbcAMP and by a specific Epac (exchange protein directly activated by cyclic AMP) agonist, 8-Cpt-cAMP. Treatment with PGE_2_ increased Rap1 activity, decreased RhoA activity and subsequently reduced the polymerization of actin by approximately 30% compared with untreated cells. In connection with this finding, polarized viral assembly platforms enriched in Gag were disrupted, altering HIV cell-to-cell transfer and the infectivity of new virions.

**Conclusions:**

Our results demonstrate that PGE_2_, through Epac and Rap activation, alters the transport of newly synthesized HIV-1 components to the assembly site, reducing the release and infectivity of new cell-free virions and cell-to-cell HIV-1 transfer.

## Background

The course of human immunodeficiency virus type-1 (HIV-1) infection is influenced by a complex interplay between viral and host factors. With the aim of controlling HIV-1 infection, the immune system triggers an inflammatory response comprising both cellular effectors and soluble factors, such as interleukin (IL)-1 and IL-6, TNF-α and interferons (IFN) α, β and γ [1]–[3]. Moreover, HIV-1 infection stimulates proinflammatory genes, including inducible nitric oxide synthase (iNOS) and cyclooxygenase-2 (COX-2) and, thus, their products NO and PGE_2_, respectively. In the immune system, PGE_2_ is mainly produced by monocytes. Several studies have reported an increase in PGE_2_ serum levels in HIV-infected patients as a consequence of viral infection and oxidative stress [4]–[6]. This lipid mediator has been shown to participate in the regulation of virus replication at different levels in a cell-type selective manner [7]. Moreover, PGE_2_ is a potent mediator of immune responses [8]–[13] that plays crucial roles in several biological events, such as T-cell proliferation and differentiation, the expression of membrane receptors, and the secretion of diverse cytokines in cellular immune reactions [14]. Therefore, PGE_2_ effects are pleiotropic, and it has been postulated that this heterogeneity depends on different pathways of PGE_2_ receptors.

PGE_2_ exerts its effects through a family of four different G protein-coupled receptors, EP1-4 [15]. Among these, the EP2 and EP4 receptors increase cyclic AMP (cAMP) via activation of adenylate cyclase (AC) [16]. To date, most cAMP-mediated effects of PGE_2_ via EP2/4 have been explained by the classic downstream target, protein kinase A (PKA), which phosphorylates downstream targets such as the cAMP response element binding protein (CREB). However some actions of cAMP have been reported to be independent of PKA [17], [18], including activation of the small GTPase Rap1. cAMP activates Rap1 through the Epac (exchange protein directly activated by cyclic AMP) protein family, which is a recently discovered family of cyclic nucleotide exchange factors (or cyclic AMP GEFs) that catalyze GTP exchange on Rap1. Epac-Rap1 signaling regulates integrin-mediated cell adhesion and chemotaxis. Thus, some reports have related the Epac-Rap1 pathway to inflammation processes and actin cytoskeleton reorganization [19], [20].

All viruses are obligate intracellular parasites without an independent metabolism that therefore strictly depend on their target cell for replication. Among other host factors, cortical actin is an important and common structure that is imperative for entry and intracellular transport of most viruses [21], [22].

HIV-1 assembly and budding take place within a lipid raft-rich platform that appears to be constrained and/or maintained by the actin and tubulin cytoskeleton. Disruption of either actin or tubulin remodeling disperses this platform, resulting in reduced cell-to-cell HIV-1 spread, and disruption of the actin cytoskeleton results in reduced cell-free virion release and viral infectivity [23]. Furthermore, some studies have reported that HIV-1 release from infected cells can be blocked by disturbing the actin network with specific toxins as cytochalasin D (cytoD) or mycalolyde B [24], [25].

In addition to infection with cell-free virions, the importance of cell-associated spread across connecting membrane bridges and close cell-cell contacts referred to as virological synapses (VSs) for HIV-1 propagation is increasingly being recognized, and it is thought to constitute the predominant mechanism of HIV-1 propagation in T lymphocyte cultures [26]–[29]. HIV-1 transmission across the VS depends on cell polarization, including dynamic reorganization of the actin cytoskeleton and recruitment of virion components to cell-cell contacts [30].

Our aim was to study the overall effect of PGE_2_ in an in vitro HIV-1-infected T cell system. Surprisingly, we showed that PGE_2_, a pro-inflammatory molecule, reduced HIV-1 replication in human PBL cultures. We ruled out the possibility that this effect was due to cytotoxicity, changes in proliferation or cell receptor expression differences. We demonstrated that this effect was mediated through the EP2 and EP4 receptors and that it was mainly dependent on the Epac-Rap1 pathway. Rap1 decreased RhoA activity, resulting in actin depolymerization. As a consequence, polarized viral assembly platforms enriched in Gag were disrupted along with virion release, infectivity and cell-to-cell transfer.

## Materials and Methods

### Cell lines

The indicator cell line TZM-bl was obtained from the NIH AIDS Research and Reference Reagent Program (Division of AIDS, NIAID, NIH: TZM-bl courtesy of Dr John C Kappes, DrXiaoyun Wu and Tranzyme, Inc.). This indicator cell line is a HeLa cell line derivative that expresses high levels of CD4 and CCR5 with endogenously expressed CXCR4. TZM-bl cells contain HIV long terminal repeat (LTR)-driven β-galactosidase and luciferase reporter cassettes that are activated by HIV tat expression. TZM-bl cells were routinely subcultured every 3 to 4 d by trypsinization and were maintained in Dulbecco's modified Eagle's medium (DMEM) (Gibco, Rockville, MD, USA) supplemented with 10% fetal bovine serum (FBS), 1% penicillin/streptomycin, and 2 mM L-glutamine at 37°C and 5% CO_2_. We obtained 293T cells from the American Type Culture Collection (Manassas, VA), and maintained them in DMEM supplemented with 10% FBS, 1% penicillin/streptomycin, and 2 mM L-glutamine at 37°C and 5% CO_2_. The CEM-T cell line was obtained from the NIH AIDS Research and Reference Reagent Program (Division of AIDS, NIAID NIH: CEM-SS (Cat 776) from Peter L. Nara). CEM-T cells and 8E5 cells, a lymphocytic leukemia line which carries one copy of the HIV genome defective for reverse transcriptase activity and produces noninfectious viral particles, were cultured in RPMI with 10% FBS, 1% penicillin/streptomycin, and 2 mM L-glutamine at 37°C and 5% CO_2_.

Human peripheral blood lymphocytes (PBL) were isolated from buffy coats from the transfusion centers of Madrid following national guidelines. Ficoll-Hypaque density gradient centrifugation (GE Healthcare, Little Chalfont, Buckinghamshire, United Kingdom)following the current procedures of Spanish HIV HGM BioBank [31]. PBL were activated over 3 d with 1 µg/ml phytohemaglutinin (PHA) and then cultured with IL-2 (60 U/ml).

Primary CD4 T-cells were isolated by immunomagnetic negative selection (MiltenyiBiotec) of PBL obtained from healthy donors. Final preparations were >95% CD4-positive T cells as assessed by flow cytometry.

### Reagents

We obtained 6-Bnz-cAMP, 8-CPT-2Me-cAMP, H89, KT5720 and AZT (3′-azido-2′,3′-dideoxythymidine) from Sigma (St Louis, MO, USA). T20 (enfuvirtide) was obtained from Roche (Palo Alto, CA). Butaprost, Misoprostol, and Sulprostone were obtained from Cayman Chemical (Ann Arbor, MI, USA). Prostaglandin E_2_ (PGE_2_), and the MEK1 kinase inhibitor PD98059 were purchased from Santa Cruz Biotechnology, Inc. (Santa Cruz, CA, USA).

### HIV-1 preparation and infection

Preparation of X4-HIV-1_NL4.3_ and R5-HIV-1_Bal_ and measurement of viral replication were performed as described (Valenzuela-Fernandez et al. 2005). Highly infectious preparations were generated by several consecutive passages of the original HIV-1 isolates in PBL. Briefly, PBL cells were infected with one synchronous dose of X4-HIV-1_NL4.3_ or R5-HIV-1_Bal_, and culture supernatants were recovered 3 d later and stored at -80°C. Freshly thawed aliquots were filtered through 0.22-µm filters before use.

HIV-1 infection was assayed in PHA-activated PBL or CEM-T cells for 2 h at concentrations of 10-30 ng X4-HIV-1_NL4.3_ p24^Gag^/10^6^ cells. Cells were washed with fresh medium to remove viral input. Infected cells were kept in culture, and p24 gag antigen was measured using a HIV-p24 ELISA kit (Innotest HIV-1 antigen mAb; Innogenetic, Ghent, Belgium) at the indicated times in each experiment.

### Production of VSV-pseudotyped and HIV-1recombinant viruses

High-titer VSV-pseudotyped recombinant virus stocks were produced in 293T cells by cotransfection of pNL4-3.Luc.R-E- together with the pcDNA3-VSV plasmid encoding the vesicular stomatitis virus G-protein using the calcium phosphate transfection system (Promega, Madison, WI, USA). Supernatants containing virus stocks were harvested 48 h post-transfection, centrifuged to remove cell debris, and stored at -80°C until use. Cell-free viral stock was tested using an enzyme-linked immunoassay for antigen HIV-p24 detection (Innotest HIV-1 antigen mAb; Innogenetic, Ghent, Belgium).

### VSV-pseudotyped HIV-1 infection assay

Cells were plated on a 24-well plate and were inoculated with virus stocks (100 ng HIV-1_NL4.3_ p24^Gag^/10^6^ cells). After 16 h, the cells were stimulated with PGE_2_ (0.1 µM) and maintained for 48 h before lysis. Then, the lysates were spun down, and the supernatant was used to measure luciferase activity with a luminometer (1450 MicrobetaLuminiscence Counter) following the instructions of the luciferase assay kit (Promega Corporation, WI, USA).

### Western blot analysis

Cells were lysed in Tris-buffered saline (TBS) containing 0.1% sodium dodecyl sulfate (SDS). The protein contents were measured using bicinchoninic acid method (BCA protein assay) according to the manufacturer's instructions (Pierce, Rockford, IL, USA). The samples were separated on a 10–15% SDS polyacrylamide gel and blotted onto a polyvinylidene fluoride membrane (Millipore, Bedford, MA, USA) by semidry transference blotting. After blocking the membranes in 10 mM TBS containing 0.1% Tween-20 (TBS-T) containing 5% skim milk, the membranes were probed with the respective primary antibodies. After washing, the membranes were incubated with HRP-conjugated secondary antibody (1∶5000 in TBS-T containing 5% skim milk) for 1 h. Proteins were detected using the Immun-Star Western C Kit (Bio-Rad Laboratories, Hercules, CA, USA). In all cases, equal amounts of total protein were analyzed across all groups. We used α-tubulin (Sigma, St. Louis, MO) as an internal control to validate the amount of protein loaded onto the gels. For quantitation, the pixel intensity for each band was determined using the Image/J program and then normalized to the amount of α-tubulin.

### TZM-bl cell line infectivity assay

TZM-bl indicator cells (2×10^4^ cells per well) were added to 96-well microtiter plate wells (Falcon, Lincoln Park, NJ) in 100 µl of complete medium and allowed to adhere for 18 h at 37°C. An equivalent amount of each virus stock (100 ng HIV-1_NL4.3_ p24^Gag^/10^6^ cells) was added to the cell monolayers in DMEM in a final volume of 100 µl. Viral infection was allowed to proceed for 2 h at 37°C, after which the cells were washed and 200 µl of complete DMEM was added. Briefly, the supernatants were removed, and the cells were lysed with a Steady Glo luciferase assay system (Promega Corporation, WI, USA). Luciferase activity was measured after 16 h. The light intensity of each well was measured on a luminometer(1450 MicrobetaLuminiscence Counter) following the instructions of the luciferase assay kit (Promega Corporation, WI, USA). Mock-infected cells were used to determine background luminescence. All of the infectivity assays were performed at least in duplicate.

### cAMP measurement

Activated and infected PBL were rinsed twice with pre-warmed serum-free medium and then pre-incubated for the indicated times with PGE_2_ (0.1 µM) at 37°C. Stimulation of the cells was stopped by the addition of 65% ice-cold ethanol. The supernatants were collected and concentrated in a Speed Vac evaporator. Intracellular cAMP concentrations were measured by commercial ELISA (cAMP Direct Biotrack™ EIA Amersham). The assay was performed according to the manufacturer's recommendation.

### Rap activation assays

Infected human CEM-T cellswere stimulated with PGE_2_ (0.1 µM) at different time points: 0, 1, 5, 15, and 30 min. Next, a Rap-1 pull-down assay was performed according to the manufacturer's recommendation (Rap1 Activation Assay Kit, Upstate Biotechnology, Lake Placid NY). The samples were subjected to SDS-PAGE and Western blot analysis using an anti-Rap1 antibody (Upstate Biotechnology, Lake Placid, NY).

### Rho assay

Rho-pull down assays using a GST-Rhotekin fusion protein were performed according to the manufacturer's recommendation (Thermo Scientific, Pierce). CEM-T cells were lysed, cell lysates were clarified by centrifugation at 18 000×*g* for 15 min at 4°C, and equal volumes of lysates were incubated with GST-RBD beads at 4°C for 45 min. The beads were washed with RIPA buffer, and the proteins associated with GST-RBD bound to beads were boiled and separated by SDS–PAGE. Immunoblotting was carried out using a specific anti-RhoA monoclonal antibody.

### Confocal analysis

Infected CEM-T cells were plated on type I collagen (PuroCol®; Inamed Biomaterials, Fremont, CA)-coated glass coverslips at 37°C for 30 min and immediately fixed in 4% paraformaldehyde and 0.025% glutaraldehyde for 10 min. The fixed cells were permeabilized with Saponine 0.1% for 10 min. After two washes in PBS, the cells were incubated with 1% BSA for 30 min and stained for intracellular Gag CA p24 using an RD1-labeled mouse anti-CA p24 monoclonal antibody (1∶100) (Beckman Coulter) and FITC-labeled Phalloidin (Invitrogen, Carlsbad, CA, USA). DAPI (1 µg/ml; Alexis Biochemicals) was applied to label nuclei. Confocal laser scanning microscopy was performed with a LEICA AOBS-TCS-SP2system. Separate images were taken in the corresponding channels, and merged images were composed. Image acquisition and data processing for all of the samples were performed under the same conditions.

### Cytotoxicity assay

Cell viability was determined using a thiazolyl blue tetrazolium bromide (MTT) (Sigma, St. Louis, MO, USA) assay following the manufacturer's instructions. Each experiment was performed in triplicate. The percentage of cell viability was calculated as a percentage of the untreated controls.

### Cell proliferation

Human PBL cells were grown in the absence or presence of PGE_2_ at different concentrations for 1 and 3 d in a 37°C and 5% CO_2_ atmosphere. Cell proliferation was measured by quantifying the bromodeoxyuridine (BrdU) incorporated into the newly synthesized DNA of the replicating cells. The assay was performed according to the manufacturer's instructions (BrdU Cell Proliferation Assay, Chemicon International).

### F-actin quantification

CEM-T cells werestimulated with PGE_2_ (0.1 µM) for different times, fixed with 4% paraformaldehyde at different time points, permeabilized with 0.5% Triton X-100 (5 min) and stained with FITC-labeled Phalloidin (Invitrogen, Carlsbad, CA, USA). The percentage of positive cells was analyzed in a FACScalibur cytometer (BD Biosciences, CA).

### Flow cytometry

Human PBL were treated with PGE_2_ for 3 d. After this, cells were harvested, washed in PBS buffer, and then stained with the following monoclonal antibodies (mAbs): CD4-PC7, CCR5-PE, CXCR4-FITC, CD69-PC5, CD38-FITC (all from BD Biosciences). After incubation with the mAbs for 30 min at 4°C in the dark, the cells were washed and fixed in 2% paraformaldehyde solution for flow cytometric analysis using a FACScalibur cytometer (BD Biosciences, CA). Live cells were gated according to their forward- and side-scatter profiles. These experiments were repeated at least three times using cells from different donors.

### Analysis of cell-free viral infections and cell-to-cell transfer by flow cytometry

To measure cell-cell spread by flow cytometry, an adaptation of the assay of Sourisseau el al. was used. Donor cells were infected with HIV-1_NL4.3_ and used a few days later when 20–40% of the cells were Gag+. Cell-to-cell HIV transfer was performed as described elsewhere[29].

To investigate the impact of PGE_2_ on cell-to-cell transfer and transmission, effector cells were treated with PGE_2_ (0.1 µM) 1 d prior to setting up co-culture. Target cells were stained with the cell trace dye CFSE Far red (CFSE) (Invitrogen, Carlsbad, CA, USA)at 37°C for 25 min, 24 h prior to starting the co-cultivation. Effector and target cells were seeded at a 2∶1 ratio to a final concentration of 0.9×10^6^ cells/ml in a final volume of 2 ml in 12-well plates, either in mixed co-culture or separated in transwell chambers with a virus-permeable membrane (0.4 µm pore size) (NUNC). An absence of CMSF-positive cells in the effector cell population confirmed the integrity of the transwell membrane. Virus transfer was assessed by flow cytometry for intracellular Gag CA p24 in target cells at 6 h after the start of co-culture.

All of the samples were analyzed using a FACS Scalibur cytometer (BD Biosciences, CA) and Kaluza software (Tree Star). The anti-CD4 mAb Leu3a (0.25 µg/ml, BD Biosciences) was used as an inhibition control.

## Results

### PGE_2_ inhibits HIV-1 replication

**Figure 1:**
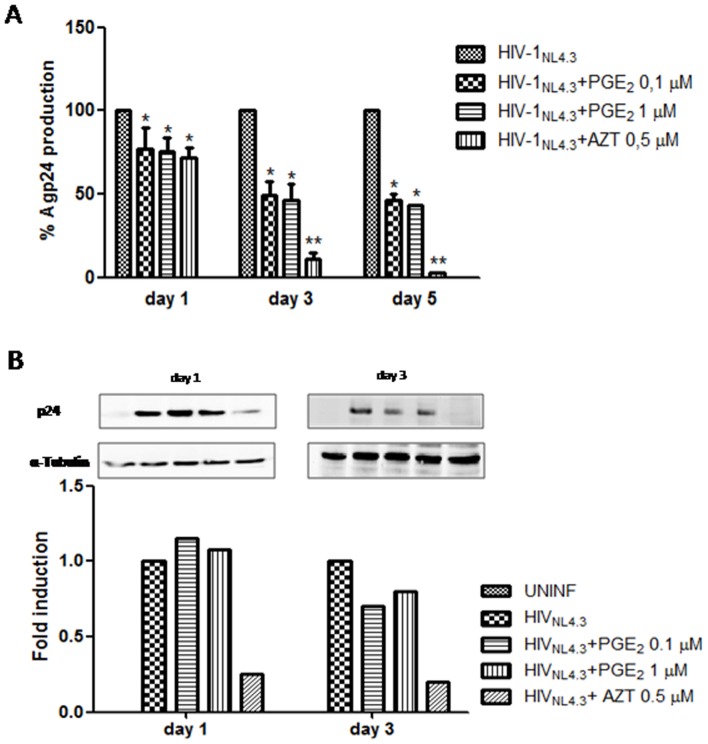
Inhibition of HIV-1 replication in human PBL by post-treatment with PGE_2_. A) Human PBL were infected with X4 strain HIV-1_NL4.3_ (20 ng HIV-1_NL4.3_ p24Gag/10^6^ cells) for 2 h and treated with PGE_2_ (0.1 and 1 µM) each 2 d. AZT (0.5 µM) was used as a positive control of inhibition. The culture supernatants were removed each 2 days and soluble p24 viral protein was monitored by ELISA at the indicted times. Results are shown as means ± SEM and expressed as a percentage of the value of the utreated control cultures. B) Representative WB for intracellular p24 protein determination in uninfected PBL or infected untreated and treated with PGE_2_ (0.1, 1 µM) or AZT (0.5 µM) at the indicated times. Antibody directed against α-tubulin was used as a protein loading control. Bottom, graph depicting the results obtained after performing a densitometer analysis of the blots. For quantification, the pixel intensity of each band was normalized to the amount of tubulin. Statistical differences in comparison to HIV-1-infected cells *:p<0.05.; **:p<0.01.

We studied the role of PGE_2_ on HIV-1 infection in PHA-activated PBL. Activated PBL were infected with X4-HIV-1_NL4.3_ (20 ng X4-HIV-1_NL4.3_ p24/10^6^ cells) for 2 h at 37°C. Cells were washed 3 times with PBS and cultured at 37°C in 5% CO_2_. PGE_2_ has a very short half-life: 9 min in human plasma and approximately 1 d in culture medium, which depends largely on the pH of the medium [32]. Therefore, the culture was supplemented with PGE_2_ every 2 d. Release of HIV p24 antigen in the supernatant of the cultures was quantified every 2 d using a p24gag ELISA kit starting at 1 d post-infection (dpi). Fig. 1A shows that the release of p24gag antigen by infected cells was inhibited by approximately 35% at 1 dpi at both doses tested, rising to 45–50% inhibition 3 d later. Similar levels of inhibition were observed at 5 dpi. Moreover, similar results were obtained with the R5-HIV-1_Bal_ isolate (Fig. S1), indicating that the effect of PGE_2_ on HIV-1 replication was independent of which HIV-1 co-receptor was used by HIV.

To determine whether the reduction of p24 gag antigen in the PBL supernatants was due to a decrease at the transcriptional or translational levels or to a deficiency in the output of new HIV, we performed Western blot experiments to quantify intracellular levels of p24 protein. As shown in Fig. 1B, at 1 dpi, no differences were found in the intracellular p24 levels between the treated and untreated PBL; but at 3dpi, the levels of viral protein were significantly lower in PGE_2_-stimulated cultures (approximately 30%) compared with controls. These results argue against the treatment with PGE_2_ affecting the transcription and translation of viral mRNA and indicate that it is necessary to observe more than one round of replication to detect changes in intracellular levels of p24 protein.

To confirm that the inhibition of HIV-1 production was not due to cytotoxic effects of PGE_2_, cytotoxicity assays were always performed in parallel with the anti-viral activity assays. The results demonstrated minimal cytotoxicity associated with PGE_2_ treatment of PBL within the concentration range of 0.1 to 1 µM at different studied times (Fig. S2A). Moreover, when we studied the proliferation of PHA-stimulated PBL treated with PGE_2_, no changes in cell proliferation were found after 3 d of culture at any PGE_2_ dose tested (Fig. S2B). However, T-cell activation involves the induction of several cell surface markers. We ruled out that the PGE_2_ effects were due to changes in cell proliferation (Fig. S2B) or alterations in the activation of cell surface markers (Fig. S2C).

### PGE_2_ does not affect viral entry, retrotranscriptase or integrase activity, or -LTR transcription

A previous study of the effect of PGE_2_ treatment on HIV-1 replication in human monocyte-derived macrophages reported that the cell surface expression of CCR5 was substantially decreased in response to treatment with 10^−5^ M PGE_2_ for 48 h [33]. Therefore, cell surface expression of CD4, CXCR4 and CCR5 in PHA-activated PBL treated or not with PGE_2_ was studied by flow cytometry after different times, and no changes were found (Fig. S2D).

**Figure 2:**
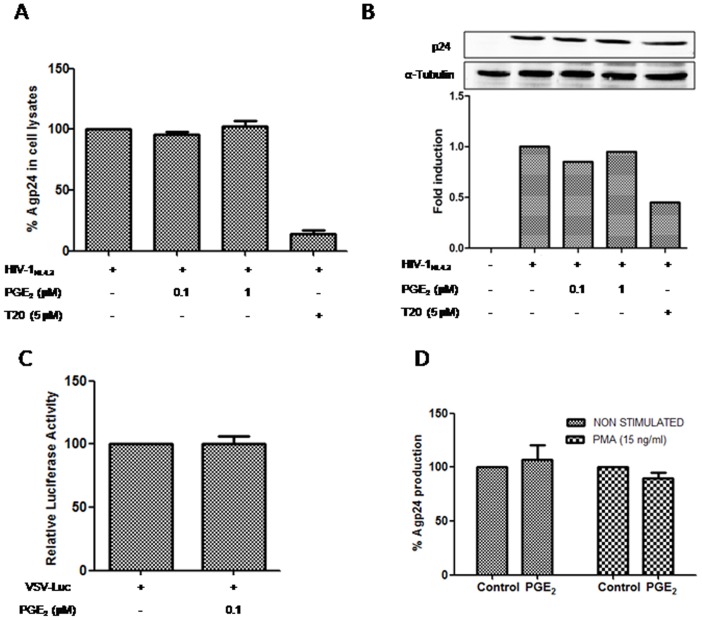
PGE_2_ does not affect initial phases of HIV-1 viral cycle. PBL were treated with PGE_2_ (0.1, 1 µM) for 16 hours, then cells were infected with HIV_NL4.3_ insolate (15 ng/10^6^ cells) for 2 h and immediately afterwards cells were lysated and p24 protein was quantified by ELISA A) and western blot B). Data represents p24 concentration relative to infected and non-treated cells (mean ± SEM) of three experiments performed in triplicate in each case. T20 (5 µM) was used as a positive control of entry inhibition. To convert band intensity into a quantitative measurement, the Western blot was analyzed densitometrically. Data present the fold induction relative to control cells C) PGE_2_ effect in post-entry steps were analyzed by infection with one-cycle viral particles VSV-pseudotyped-pNL4-3.Luc (100 ng HIV-1_NL4.3_ p24^Gag^/10^6^ cells). Data are shown as percentage of non treated cells. Results are from three independent experiments performed in triplicate. D) To study PGE_2_ effect in LTR-transactivation, 8E5/LAV were used. Cells were activated or not with PMA (15 ng/ml) and treated with PGE_2_ for 3 days. The culture supernatants were removed and p24 viral protein was monitored by ELISA. All data are shown as percentage of non treated cells.

To further analyze the involvement of PGE_2_ in viral entry, PBL were pre-incubated with PGE_2_ (0.1 or 1 µM) for 16 h and infected with HIV (X4-HIV-1_NL4.3_) for 2 h. Then, cells were washed and lysed, and p24 levels were measured using a p24gag antigen ELISA kit and by Western blotting. Analysis of the intracellular viral protein levels failed to reveal any gross changes in p24 expression between treated and non-treated HIV-1-infected PBL (Figs. 2A, 2B). Shortly after the viral capsid enters a cell, the reverse transcriptase enzyme (RT) liberates the single-stranded (+) RNA genome from the attached viral proteins and copies it into a cDNA molecule [34]. Subsequently, the DNA is transported to the nucleus, where it is spliced into the human DNA by the HIV enzyme integrase [35]. To address the role of PGE_2_ in RT activity and viral DNA integration, we used a single virus infection cycle with HIV-1 VSV-Luc, which enters cells via endocytosis and thereby bypasses CD4/CXCR4 and the cortical actin. We infected the PBL with a VSV-pseudotyped HIV-1 (100 ng X4-HIV-1_NL4.3_ p24/10^6^ cells). High luciferase activity levels were detected 2 d after PBL infection with the VSV-pseudotyped HIV-1 clone, and treatment with 0.1 µM of PGE_2_ did not alter luciferase activity (Fig. 2C). Summing up, PGE_2_ did not affect the RT enzyme or the level of viral DNA integration into the cell genome.

Previous reports have shown that PGE_2_ treatment of T-cell cultures increases viral LTR transcriptional activity through c/EBP and CREB [36] or NF-κB [37]. To determine the role of PGE_2_ in LTR transcriptional activity, we used the 8E5 cell line containing a single integrated defective copy of HIV-1 [38]. The 8E5 cells were exposed or not to PMA (15 ng/ml) alone or in combination with PGE_2_, and the supernatants were assayed for HIV p24 core antigen at 3 d. As previously reported [39], a 10-fold increase in p24 gag antigen was observed in PMA 8E5 treated cells (Fig. S3). The 8E5 cells stimulated or not with PMA and treated with PGE_2_ did not modify p24 gag levels compared to non-PGE_2_-treated cells (Fig. S2D). Summing up, PGE_2_ did not modify LTR transcriptional activity in 8E5 cells.

### Repression of HIV-1 production, viral infectivity and cell-to-cell spread by PGE_2_

**Figure 3:**
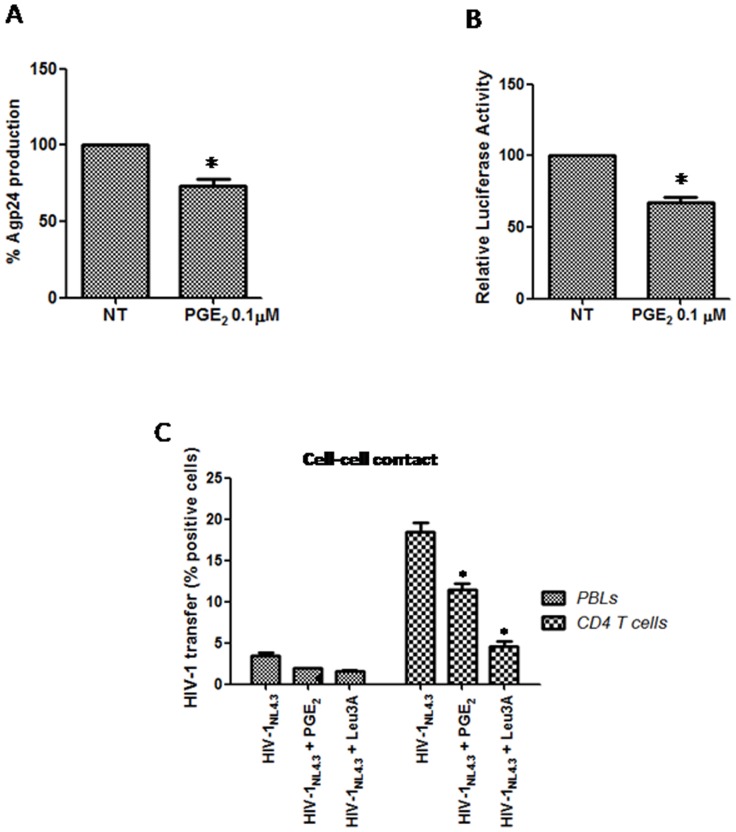
Effects of PGE_2_ on HIV-1 release and viral infectivity. A) HEK 293T cell line was transfected with pNL4.3-Luc-R-E- and CXCR4-tropic env glycoprotein vector, and treated with PGE_2_ (0.1 µM) 2d. Agp24 levels in supernatants were quantified by ELISA. B) To study the infectivity of new HIV virus the TZM-bl cells were incubated with the supernatants from PBL infected and treated or not with PGE_2_ (0.1 µM) as detailed in Materials and Methods. After 16 h, the cells were lysed and luciferase activity was determined. Results are shown as percentage of non treated. C) PBL or purified primary CD4 T cells were infected with HIV-1_NL4-3_ isolate and cultured during 2 d. Bellow cells were treated with PGE_2_ 1 d more, and then co-cultured with the CMSF labeled target cells in presence or not of PGE_2_. Effectors and target cells were seeded at a 2∶1 ratio in mixed co-culture. Anti-CD4 antibody Leu3a (0.25 µg/ml) was used as positive control. Virus transfer was assessed by flow cytometry for intracellular Gag CA p24 in target cells at 6 h after the start of co-culture. Results represent the means of 3 individual experiments. Error bars indicate standard error values. Statistical differences in comparison to control values (A, B) or shown as a percentage of positive cells for HIV-1 transfer ± SEM, *:p<0.05.

All of the results pointed to a role for PGE_2_ in the late steps of the HIV-1 life cycle. To study the biological relevance of the repressive action of PGE_2_ on HIV-1 release, we researched the effect of PGE_2_ on HIV-1 production. We cotransfected 293T cells with pNL4-3.Luc.R-E- (60 µg; NIH-AIDS reagent program 6070013) and a CXCR4-tropic *env* glycoprotein vector (30 µg; HXB2-env; NIH-AIDS reagent program 5040154), and the cells were stimulated or not with PGE_2_. The virus production was quantified after 2 d using a p24gag antigen ELISA kit. The viral production in 293T cells treated with PGE_2_ was significantly lower than production in untreated cells (Fig. 3A). Our result was consistent with PGE_2_ exerting its effect late in the HIV-1 life cycle, reducing virus release into the culture supernatants.

To further probe the inhibitory mechanism of PGE_2_, we used a virus-cell infection system that utilizes an indicator cell line, TZM-bl, which has been engineered to express CD4 and CCR5. Because CXCR4 is endogenously expressed on TZM-bl cells, it is susceptible to infection by diverse HIV-1 isolates. For the infectivity assay, supernatants from PBL were normalized for p24gag content (100 ng X4-HIV-1_NL4.3_ p24/10^6^ cells) and tested on TZM-bl cells. Interestingly, supernatants from HIV-1-infected PBL treated with PGE_2_ produced a decrease in luciferase activity of approximately 30% compared with supernatants from untreated cells (Fig. 3B). To summarize, our results indicate that PGE_2_ decreases both the viral release and the infectivity of cell-free viral particles.

Because cell-cell virion transfer constitutes the predominant mechanism of HIV-1 propagation in T lymphocyte cultures, we directly compared the efficiency of cell-cell transfer in the presence or absence of PGE_2_. Thus, we performed a flow cytometry assay to quantify the appearance of Gag^+^target T cells following co-culture with infected T cells. PBL or purified primary CD4^+^ T cells were infected with 400 ng/10^6^ cells and cultured for 48 h, resulting in 10-12% infection rate as determined by measurement of the intracellular p24 expression using flow cytometry. Thereafter, the cells were treated with PGE_2_ for 24 h more, and then co-cultured with uninfected cells that were previously incubated with CMSF (see M&M) at a 2∶1 infected: uninfected cell ratio, which has been reported to increase viral spread [29]. The cells were then co-cultured to allow cell-cell contact or separated by a small pore-size transwell membrane (0.4 µm) that excluded cells while permitting free viral diffusion. After 6 h, viral CA p24 was detected in the target cells using flow cytometry, indicating viral transfer. The results showed that when PGE_2_ was present in the mixed co-cultures, the transfer was decreased when using PBL or purified primary CD4^+^ T cells (Fig. 3D). Addition of the blocking anti-CD4 antibody Leu3a abrogated the capture of p24 antigen. Nevertheless, no differences in CA p24 were detected after 6 h in any of the potential target cell populations in the transwell co-culture experiments, where direct cell-cell contact was blocked, confirming that PGE_2_does not affect the transmission of free virus, but rather it is more involved in cell-cell viral transmission.

### PGE_2_ decreases actin polymerization

**Figure 4:**
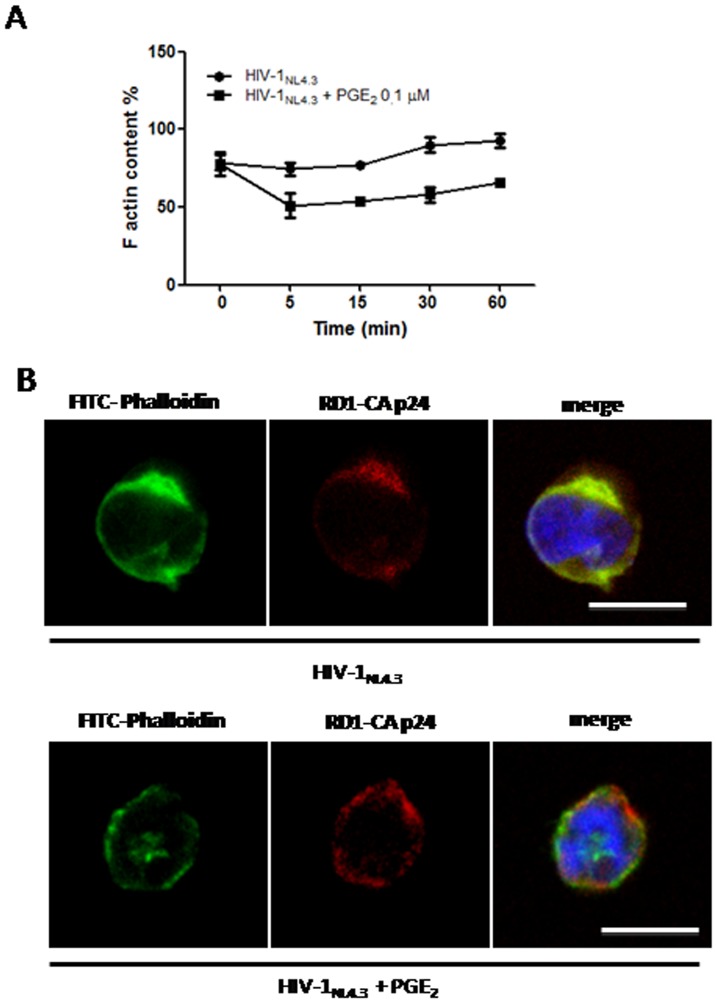
Effects of PGE_2_ treatment on actin poymerization and Gag distribution in T cell. Actin polymerization triggered by PGE_2_ (0.1 µM) for the indicated times in HIV-1 infected cells. Data are from three independent experiments, presented as mean ±SEM. D) Actin polimerization and Gag distribution in a T cell line treated or not with PGE_2_. Infected CEM-T cells treated or not with PGE_2_ (0.1 µM) for 16 h, were stained with FITC-labeled Phalloidin, and RD1-labeled mouse anti-CA p24 monoclonal antibody, in order to visualize actin organization and Gag distribution, respectively. Scale bar: 5 µm.

Our results suggest that PGE_2_ acts in the late stages of the HIV life cycle. Moreover, some studies have indicated that actin may be involved in these final stages such as the viral assembly and budding processes [40]–[43] and in HIV cell-cell transmission [44]–[46]. Thus, we researched whether reorganization of the actin cytoskeleton was affected after PGE_2_ treatment in T cells. Infected CEM cells were stimulated with PGE_2_ (0.1 µM) at the indicated times, and polymerized actin was evaluated using flow cytometry (see M&M). As shown in Fig. 4A, PGE_2_ treatment reduced actin polymerization in HIV-infected cells compared with untreated cells at all of the times indicated. Moreover,virion budding and release can be affected by disruption of the HIV-1 polarized assembly platform. Therefore, in addition to actin polymerization, we investigated the intracellular distribution of Gag under PGE_2_ treatment. In infected CEM cells that were fixed, permeabilized, and stained, Gag was localized at one pole of the cell. As expected, treatment of CEM cells with the actin-depolymerizing agent cytochalasin D (10 µM) significantly reduced the percentage of cells with capped Gag (data not shown). Surprisingly, PGE_2_ treatment resulted in a loss of polarized Gag staining in CEM cells (Fig. 4B).

### PGE2 decreases Agp24 production via the EP2 and EP4 receptors

**Figure 5:**
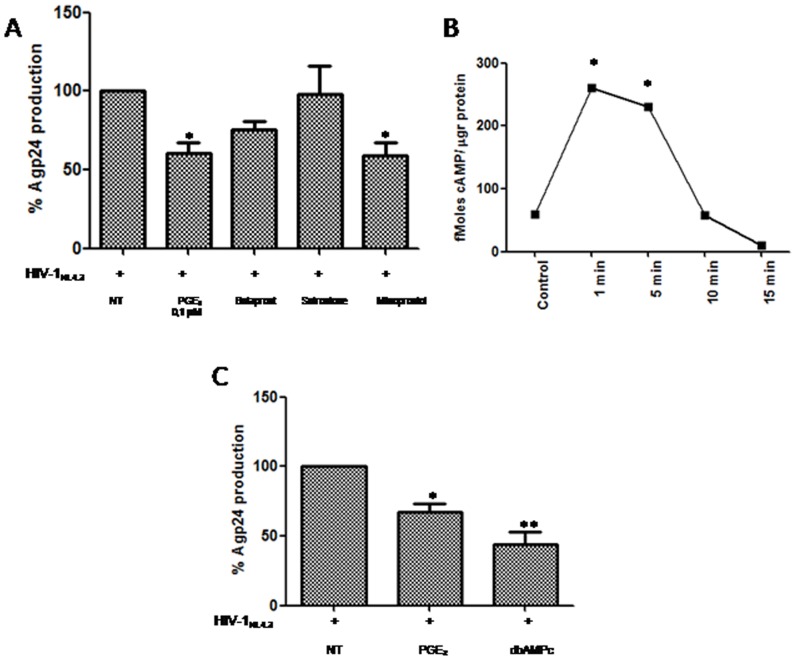
Role of different EP receptors and cAMP on HIV-1 repression. A) Infected PBL were treated with different agonists: butaprost (EP2) (10 µM), sulprostone (EP1/EP3) (10 µM) or misoprostol (EP4, EP3>EP2) (35 µM), or. Agp24 levels were quantified by ELISA 3 d later. B) PGE_2_ interaction with EP2/EP4 receptors induces cAMP liberation. PBL were treated with PGE_2_ (0.1 µM) for the indicated times, and intracellular cAMP was quantified as described under “Material and Methods”. C) Effect of dbcAMP (200 µM) on Agp24 production in human PBL after 3 days of infection. Statistical differences in comparison to HIV-1-infected (A,C) or control cells (B) *:p<0.05.; **:p<0.01.

To determine which PGE_2_ (EP) receptors were involved in the decrease of HIV-1 replication in human PBL, we employed synthetic analogues of PGE_2_ that act as selective EP receptor agonists. We used three EP receptor agonists: butaprost, an EP2-specific agonist; misoprostol, a preferential EP3/EP2 agonist that also binds EP4 at high concentrations; and sulprostone, an EP1/EP3 agonist. Misoprostol and butaprost induced a decrease in p24gag antigen release in comparison to PGE_2_, whereas sulprostone did not produce this effect (Fig. 5A). These results indicated that the inhibitory effect of PGE_2_ on HIV-1 replication in PBL cultures was mediated through EP2 and possibly EP4 receptors. Because EP2 receptors are coupled to Gα resulting in the activation of adenylatecyclase, we analyzed whether PGE_2_ induced cAMP liberation in PBL. As shown in Fig. 5B, PGE_2_ treatment produced a rapid increase in cAMP as early as 1 min after stimulation followed by a gradual decrease. In addition, the effects of PGE_2_ on viral production were mimicked by dbcAMP, suggesting that cAMP is an important mediator of PGE_2_ effects (Fig. 5C).

### PGE_2_ effect on HIV-1 replication involves Epac/Rap/RhoA signaling

**Figure 6:**
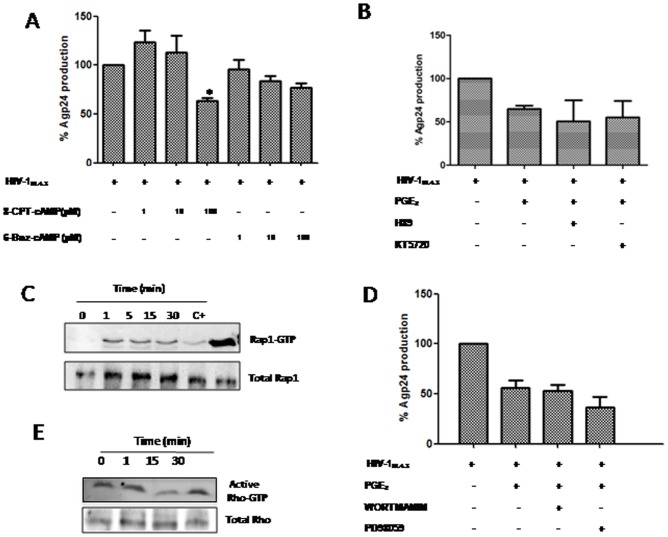
PGE_2_ regulates T cell actin polymerization throughout Epac/Rap1 and RhoA signaling. A) PBL were infected and cultured in the presence of different concentrations of 8-CPT-2-OMe-cAMP (1, 10, 100 µM) or 6-Bnz-cAMP (1, 10, 100 µM) for 3 d. Supernatants were collected and Agp24 levels were determined by ELISA. B) Effect of PKA inhibitors (H89 5 µM, KT5720 200 nM) on PBL treated with PGE_2_ 3d post-infection. C) Pull-down assay of active Rap on infected CEM-T cells for the indicated times. Western blots were probed for Rap1. D) Effect of PI3k and ERK1 inhibitors, wortmanin (100 nM) and PD98059 (10 µM) respectively, on HIV-1 replication in PGE_2_-stimulated PBL. E) Immunoblot analysis of pull-down assay for Rho-GTP. Rho-A antibody was used to detect activated Rho-GTP from a pull-down assay using beads covalently bound to Rhotekin and also total Rho-A in cell lysates. Error bars indicate standard error values. Statistical differences in comparison to non-treated HIV-1-infected cells *:p<0.05.

Intracellular cAMP regulates diverse cellular functions, mainly through two downstream effectors: Epac and PKA. To evaluate the effects of both effectors on cAMP-induced HIV-1 inhibition, we treated PBL cultures with different concentrations of two highly selective cAMP analogs to activate PKA or Epac, 6-Bnz-cAMP and 8-pCPT-2-*O*-Me-cAMP, respectively. The results demonstrated that 6-Bnz did not significantly reduce p24gag antigen release; however, higher doses of 8-CPT (100 µM) decreased viral replication approximately 40%, confirming the main role of Epac protein on the effects of PGE_2_ as detailed above (Fig. 6A). To rule out the involvement of PKA on PGE_2_ actions in our model, the effects of PKA inhibitors such as H89 and KT5720 were researched. Surprisingly, no inhibitor affected p24 gag antigen production in PGE_2_-stimulated PBL (Fig. 6B), confirming again the main role of Epac. To confirm the partial involvement of this signaling on PGE_2_-mediated effects, we performed new western blot assays with longer incubation times, which resulted in a moderate increase in p-CREB activity in treated cells at the indicated times (Fig. S5A).

In summary, Epac activation by cAMP is the main pathway responsible for the inhibition of HIV-1 production.Epac proteins mediate cAMP-dependent activation of Rap. Therefore, the requirement for Rap activation in this process was evaluated. Rap activation was measured in PBL treated with PGE_2_ for different times. PGE_2_ treatment resulted in a rapid Rap activation as confirmed by pull-down experiments (Fig. 6C), indicating that this protein could also be involved in the repression of HIV-1 replication. Alternatively, ERK signaling is initiated by small GTPases such as Rap, which is a member of the Ras family. Several studies have indicated that cAMP-dependent signaling is closely interwoven with the phosphatidyl inositol 3-kinase (PI3K)/PKB/Akt pathway [47], [48]. When assessing the involvement of ERK in PGE_2_-mediated HIV-1 inhibition, we did not observe an overall statistically significant effect of the inhibitor PD98059 on p24gag antigen production (Fig. 6D). Furthermore, to check the involvement of PI3K signaling in PGE_2_-mediated HIV-1 inhibition, we tested the PI3K inhibitor, wortmannin, which is a highly potent and selective PI3K inhibitor [49]. As expected, this drug did not show a significant effect on HIV-1 infection (Fig. 6D). In addition, pAkt levels did not increase after treatment at the indicated times, indicating that there was no involvement of these pathways in the observed effects of PGE_2_ on HIV-1 replication (Fig. S5B).

We have previously shown a reduction in actin polymerization in HIV-1- infected CEM cells treated with PGE_2_ (Fig. 4A). Moreover, it has previously been reportedthat Rap1 negatively regulates Rhoin other systems[50], and it is clear that Rho controls actin stability. Because we found that PGE_2_ induces Rap1 activation and inhibition of actin polymerization, we sought to study Rho activation. We observed that PGE_2_ treatment of CEM cells produced decreased RhoA activity (Fig. 6E).

## Discussion

Virus infections often cause acute inflammatory responses that are mediated by several cellular effectors and soluble factors. Although these responses have an important protective role, they may also have deleterious effects on the host. The balance between these protective and deleterious effects may ultimately determine the course of disease after viral infection. PGs are important regulators of this inflammatory reaction.

PGs serve as second messengers that elicit a wide range of physiological responses in cells and tissues. In particular, the PGs of the E series are known to have immunomodulatory properties. In addition to mediating inflammatory symptoms, PGs may exert anti-inflammatory effects. For example, PGE_2_ inhibits the secretion of IFN-γ, a cytokine that has antiviral activity [51], and switches the immune response toward a Th2-type cytokine profile (IL-4 and IL-5), which are less effective in developing an antiviral response [9]. In addition, a direct role for COX and PGs in controlling viral replication has been described for a wide range of virus infections, but their actions appear to be dependent on both the virus and cell type [52]. For instance, COX and/or PG is required for efficient replication of herpes viruses [53], [54], bovine leukemia virus [55], and rotavirus [56]. In the case of HIV-1, PGE_2_ has been shown to stimulate virus replication by activating viral promoters [36]. It has been described that PG negatively affects HIV replication in macrophages [7]. However, the mechanisms by which PGs regulate viral replication are largely unclear.

We have examined how PGE_2_ treatment affects HIV infection. We have found a decrease (35–40%) in p24 Ag levels after a single post-infection PGE_2_ treatment (data no shown).Repetitive dosing every 2 d increased the inhibition values of p24 Ag obtained 3 dpi from an initial 35% to 55% or even 60% in the case of the highest PGE_2_ dose (1 µM). As described above, PGE_2_ has a very short half-life [32]. Therefore, it is logical to find an increase of the inhibitory effect on p24Ag production after repetitive dosing. However, the results obtained after 5 d of infection were very similar to those found 3 d post-infection, indicating that the maximum inhibition was reached after 3 d of treatment. Moreover, the inhibitory effect of PGE_2_ on viral replication was independent of the viral isolate used, indicating a viral co-receptor type independency. One of the most interesting aspects was the discrepancy between the results of p24 Ag quantification in the culture supernatant of PBL and in cell lysates at 1 dpi. These data indicated that PGE_2_ affected HIV production and not “de novo” synthesis of p24 Ag.

PGE_2_ is well known to regulate cell functions through cAMP. It has been shown that cAMP inhibits cell proliferation and reduces the effector functions in T-cells [57]. Our results show that HIV-1 inhibition mediated by PGE_2_ is not due to a change in cell proliferation. However, it has been reported that cAMP may stimulate cellular proliferation by acting synergistically with other growth factors. The mitogenic effects of cAMP are PKA and Epac-dependent and involve modulation of downstream signaling networks essential for proliferation [58], [59]. The differences between the results of different studies might be due to different cell models used as well as the time of treatment.

A previous study of the effect of PGE_2_ treatment on HIV-1 replication in human PBMCs reported that the cell surface expression of CCR5 was substantially decreased in response to treatment with 10^−5^ M PGE_2_ for 2 d. The observed decrease in HIV-1 replication in that study was attributed to the decrease in the CCR5 co-receptor expression and was interpreted to mean that HIV-1 entry into the cells was decreased [33]. Here, we ruled out any similar effect because we did not find any modulation of surface CD4, CXCR4 or CCR5 levels after PGE_2_ treatment.

Initially, the effects of cAMP were solely attributed to activation of PKA and cAMP-gated ion channels, but the contribution of the alternative cAMP target Epac has become increasingly appreciated (reviewed in [60]. Although some cellular responses associated with intracellular cAMP increase are regulated exclusively by the PKA [61]or Epac [62] signaling pathways, in many processes, they are frequently interconnected [63] or can even play opposing roles [64]. The synthesis and characterization of cAMP analogs that selectively bind and activate either PKA or Epac have now made it possible to discriminate between the two signaling pathways [65].

Epac proteins were initially characterized as guanine nucleotide exchange factors (GEF) for both Rap1 and Rap2 [66]. Rap belongs to the Ras family of small G proteins, which cycle between an inactive guanosine diphosphate (GDP)-bound state and an active guanosine triphosphate (GTP)-bound state. Many effectors proteins, including adaptor proteins implicated in the modulation of the actin cytoskeleton, regulators of G proteins of the Rho family and phospholipases (reviewed in [60]) to relay signaling downstream from Rap. Moreover, Rap1A positively regulates T-cell signaling, and these effects have been reported to be mediated via the activation of integrins [67], [68]. We have demonstrated that PGE_2_ is able to increase cAMP levels. Our findings are consistent with the fact that the effects of PGE_2_ are predominantly mediated through Epac/Rap1 rather than through PKA. Our evidence that the observed effect of PGE_2_ is independent of PKA is based on our findings that the specific PKA inhibitors used did not produce any increase inHIV-1 replication. Moreover, the specific analog 6-Bnz-1 at 100 µM was indeed unable to reduce Agp24 production to the same level as 8-CPT did. PGE_2_ can exert its actions through one of several signaling pathways, depending on the receptor subtype through which the cellular response is mediated. Our results show that ERK and PI3K inhibitors are unable to reverse the inhibitory PGE_2_ effectof HIV-1 replicationin human T cells.

Within the HIV life cycle, we can differentiate several stages: entry, reverse transcription of the viral genome, integration of proviral DNA, genome transcription to give rise to new copies of RNA and new proteins for viral assembly and exit of new viral particles [69]. In recent years, a great variety of proteins and cellular factors involved in each of the stages of the cycle have been described that are therefore capable of altering HIV-1 replication [70]. PGE_2_ is one of the host factors that has been described as a modulator of the HIV cycle. However, there is little data about PGE_2_ related to a decrease of viral replication [7], [33] given that our results are the first to elicit this effect in T cells. In our study, the screening of the viral cycle stages discounted PGE_2_ involvement in the processes of entry, reverse transcription, integration, viral transcription and translation. All of our results pointed to a role for PGE_2_ in the late stages of the HIV-1 viral cycle such as viral assembly or budding. To elucidate the effect of PGE_2_ on viral assembly, we performed confocal microscopy experiments to analyze the Gag distribution in CEM-T cells. Microscopic examination of Gag in untreated cells revealed co-localization to a polarized F-actin distribution. PGE_2_ treatment (0.1 µM) changed F-actin and Gag delivery patterns; both staining patterns were redistributed and dispersed around the inner leaflet of the plasma membrane and within the cytoplasm. These results indicated a possible role of PGE_2_ in actin polymerization and distribution that was confirmed by our flow cytometry experiments, which showed a decrease in the number of F-actin-positive cells in PGE_2_-treated CEM-T cells. Some studies have shown the involvement of actin in the assembly and output of new HIV progeny by the use of actin polymerization inhibitors [24], [25]. The mechanism of transport of HIV-1 Gag and Env to the site of virion assembly is a directed process that is currently under intense scrutiny. It is known that F- actin is associated with the nucleocapsid (NC) domain of the polyprotein Gag, thus allowing its transport to assembly areas [40]–[43]. A recent article reported that the HIV maturation process takes place next to the plasma membrane where there is the greatest concentration of Gag and Gag/Pol polyprotein. Thus, the changes in Gag location and differences in the Gag/Gag-Pol ratio modify the infectivity of the new viral particles [71]. Our data showed a significant reduction in the output process of new viral particles (30%) and a significant decrease in the infectivity of the new viral particles (40%). Taken together, and using all of our data, we could attribute the decrease in infectivity to a diminished Gag concentration during viral assembly and at the maturation sites, leading to fewer p24-gag molecules per virus; in further studies, experiments will be performed to confirm this hypothesis.

In addition to infection with cell-free virions, the importance of cell-to-cell transmission in HIV-1 propagation, referred to as VS, is increasingly recognized and it is thought to constitute the predominant mechanism of HIV-1 propagation in T-lymphocyte cultures [26]–[29], [72], [73]. HIV-1 transmission across the VS depends on cell polarization, including the dynamic reorganization of the actin cytoskeleton and recruitment of virion components to cell-cell contacts [30], [41], [74]. Thus, we researched the impact of PGE_2_ treatment on HIV cell-to-cell transfer, obtaining a significant decrease in the viral transfer of PGE_2_-treated cells compared with untreated cells. Our data described above show a decrease in actin polymerization in the presence ofPGE_2_; these data are in agreement with the data published by Clare Jolly, who showed that cell treatment with actin polymerization inhibitors decreased the number of VS as a consequence of the inhibition of Gag and Env capping.

In summary, we demonstrated that PGE_2_ triggered the inhibition of HIV through Epac and Rap-1. The activation of these proteins altered the actin-dependent transport of newly synthesized HIV-1 components to the assembly platform site, and this disruption reduced HIV-1 transmission, cell-free virion release and viral infectivity. Several studies have reported an increase in PGE_2_ serum levels in HIV-infected patients as a consequence of viral infection and oxidative stress [4]-[6]. Although in patients HIV-1 viral load depends on multiple factors, it would be required to study differences in HIV-1 viral load as consequence of their PGE2 levels in serum.

## Supporting Information

Figure S1 **Inhibition of HIV-1_Bal_ replication in human PBL by post-treatment with PGE_2_.** Human PBL were infected with R5 strain HIV_BaL_ (15 ng/10^6^ cells) for 2 h and treated with PGE_2_ (0.1, 1 µM) for 3 days. AZT (0.5 µM) was used as a positive control of inhibition. HIV-1 infection was monitored by measuring Agp24 production in supernatants by ELISA at 3 d. Results are shown as mean ± SEM and expressed as a percentage of the value of the untreated control cultures. Statistical differences in comparison to HIV-1-infected cells *:p<0.05.; **:p<0.01.
(TIF)

Figure S2 **PGE_2_ effects are not due to changes in cell viability, proliferation, activation or cell receptors.** A) Human activated PBL were treated with PGE_2_ at doses of 0.1, 1 µM, and cell viability was measured by MTT assay 3, 5, and 7 d later. B) Human activated PBL were exposed to 0.1, 1 µM of PGE_2_, and 3 d later cell proliferation was measured by incorporation of BrdU. C) Expression of CD69, and CD38 after PGE_2_ treatment at the indicated times. D) Activated PBL were treated with PGE_2_ (0.1 µM), and CD4, CXCR4 and CCR5 surface expression was evaluated by flow cytometry at the indicated times. The experiments showed are the mean of three independent experiments. Live cells were gated according to forward and side scatter profiles. Results represent the means of 3 individual experiments. Error bars indicate standard error values.
(TIF)

Figure S3 **Effect of PMA in LTR-promoter expression.** PMA treatment for 3 d increase p24 core protein levels in 8E5 cell line culture supernatants about 10 times.
(TIF)

Figure S4 **HIV free-viral particle.** Purified primary CD4 T cells were infected during 3 d with HIV-1_NL4-3_ isolate, treated with PGE_2_, and then co-cultured either with the CMSF labeled target cells. Effector and target cells were seeded at a 2∶1 ratio separated in transwell chambers with a virus-permeable membrane (0.4 µm pore size). Virus transfer was assessed by flow cytometry for intracellular Gag CA p24 in target cells at 6 h after the start of co-culture. Results are shown as a percentage of positive cells for HIV-1 transfer ± SEM of 3 independent experiments.
(TIF)

Figure S5 **Western blot of A) p-CREB and B) p-AKT in PGE_2_-stimulated CEM-T cells at the indicated times.** Bottom, the graph depicting the results obtained after performing a densitometer analysis of the blots. Western blot representative of three is shown.
(TIF)
