# Supplementary figures and images for: Prostaglandin E2 Reduces the Release and Infectivity of New Cell-Free Virions and Cell-To-Cell HIV-1 Transfer
Source: PLoS One. 2014 Feb 25;9(2):e85230. doi: 10.1371/journal.pone.0085230 (PMC3934822; doi:10.1371/journal.pone.0085230)

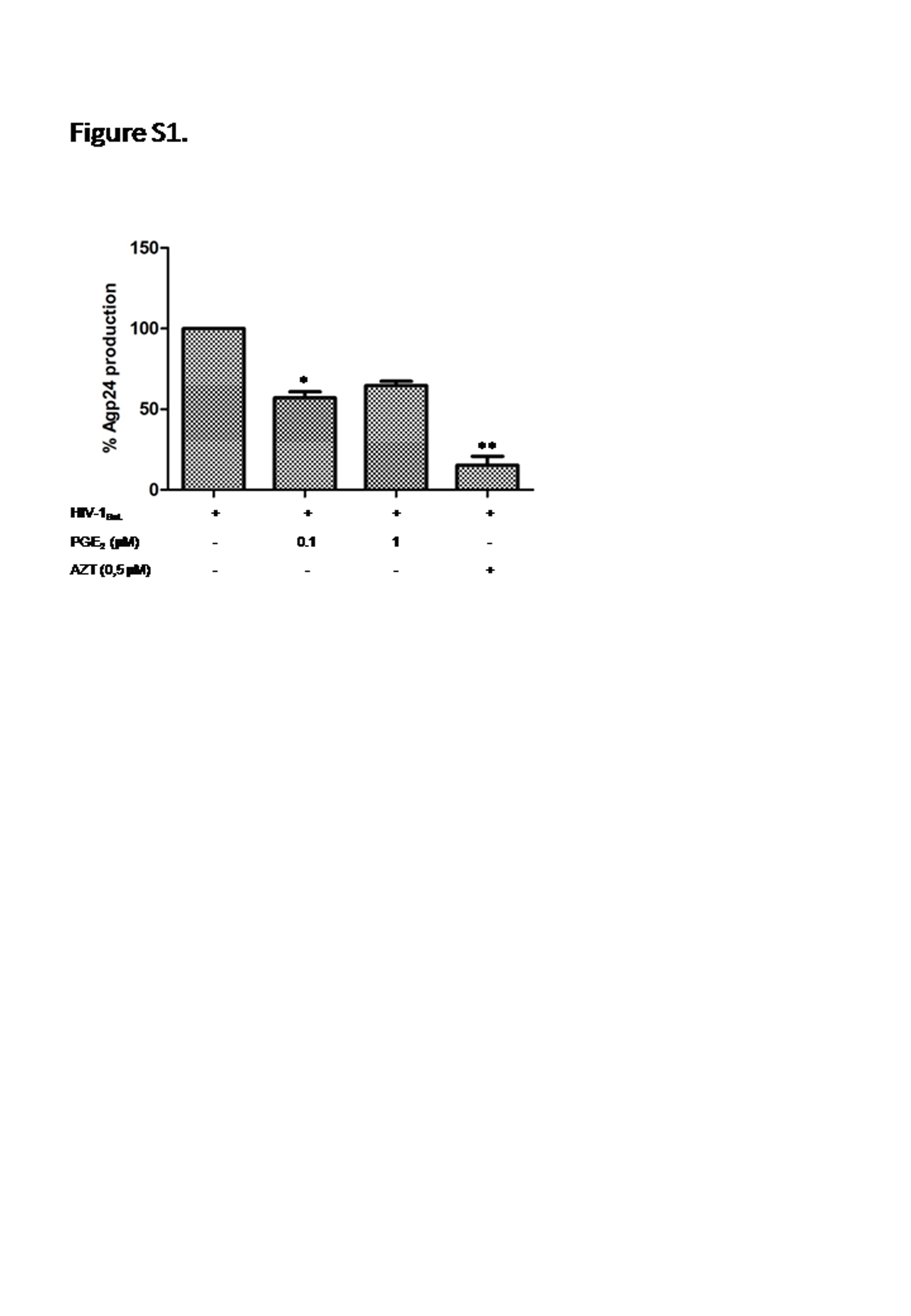

Supplement: Figure S1 — Inhibition of HIV-1Bal replication in human PBL by post-treatment with PGE2. Human PBL were infected with R5 strain HIVBaL (15 ng/106 cells) for 2 h and treated with PGE2 (0.1, 1 µM) for 3 days. AZT (0.5 µM) was used as a positive control of inhibition. HIV-1 infection was monitored by measuring Agp24 production in supernatants by ELISA at 3 d. Results are shown as mean ± SEM and expressed as a percentage of the value of the untreated control cultures. Statistical differences in comparison to HIV-1-infected cells *:p<0.05.; **:p<0.01. (TIF) [file pone.0085230.s001.tif]

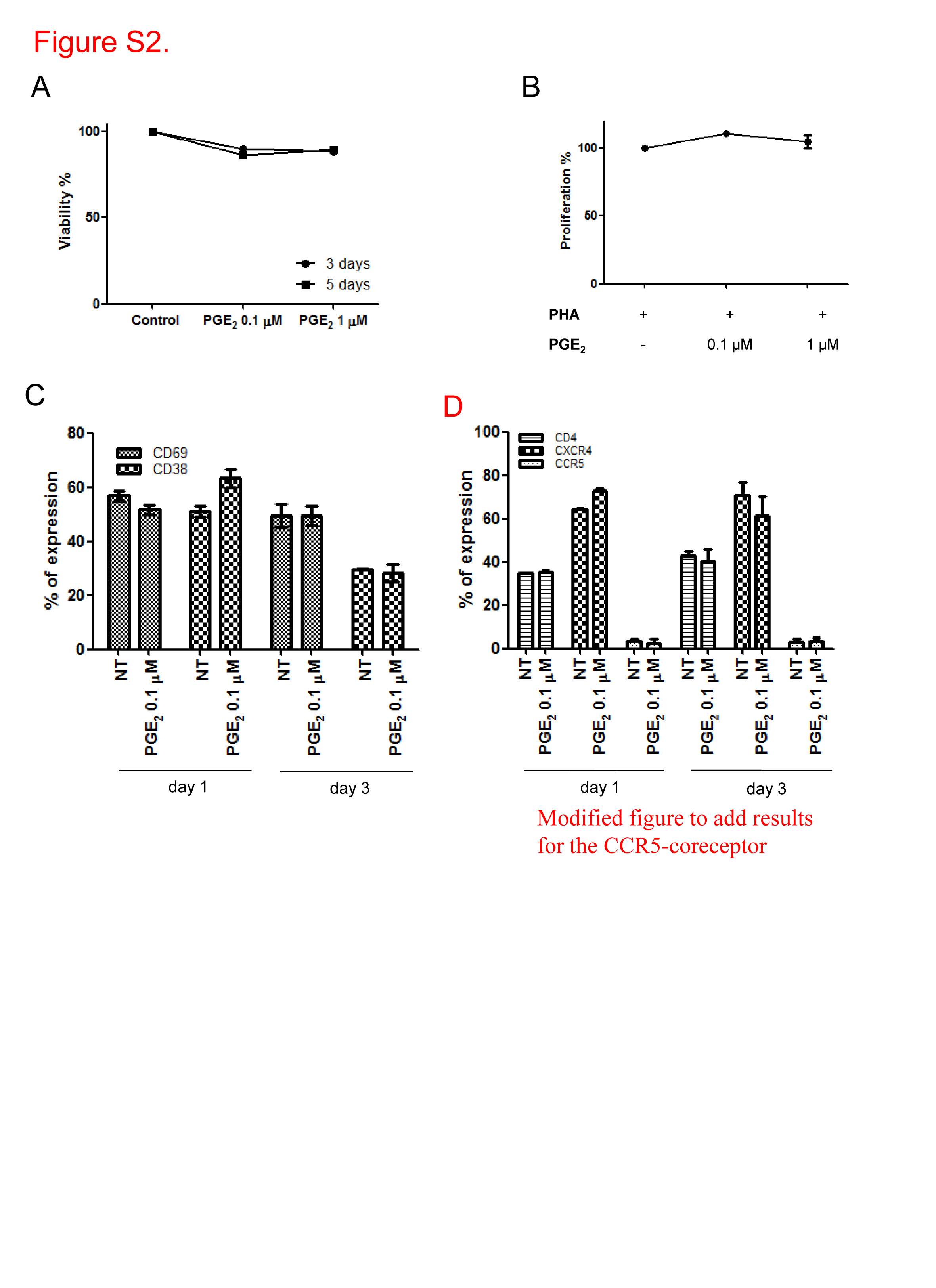

Supplement: Figure S2 — PGE2 effects are not due to changes in cell viability, proliferation, activation or cell receptors. A) Human activated PBL were treated with PGE2 at doses of 0.1, 1 µM, and cell viability was measured by MTT assay 3, 5, and 7 d later. B) Human activated PBL were exposed to 0.1, 1 µM of PGE2, and 3 d later cell proliferation was measured by incorporation of BrdU. C) Expression of CD69, and CD38 after PGE2 treatment at the indicated times. D) Activated PBL were treated with PGE2 (0.1 µM), and CD4, CXCR4 and CCR5 surface expression was evaluated by flow cytometry at the indicated times. The experiments showed are the mean of three independent experiments. Live cells were gated according to forward and side scatter profiles. Results represent the means of 3 individual experiments. Error bars indicate standard error values. (TIF) [file pone.0085230.s002.tif]

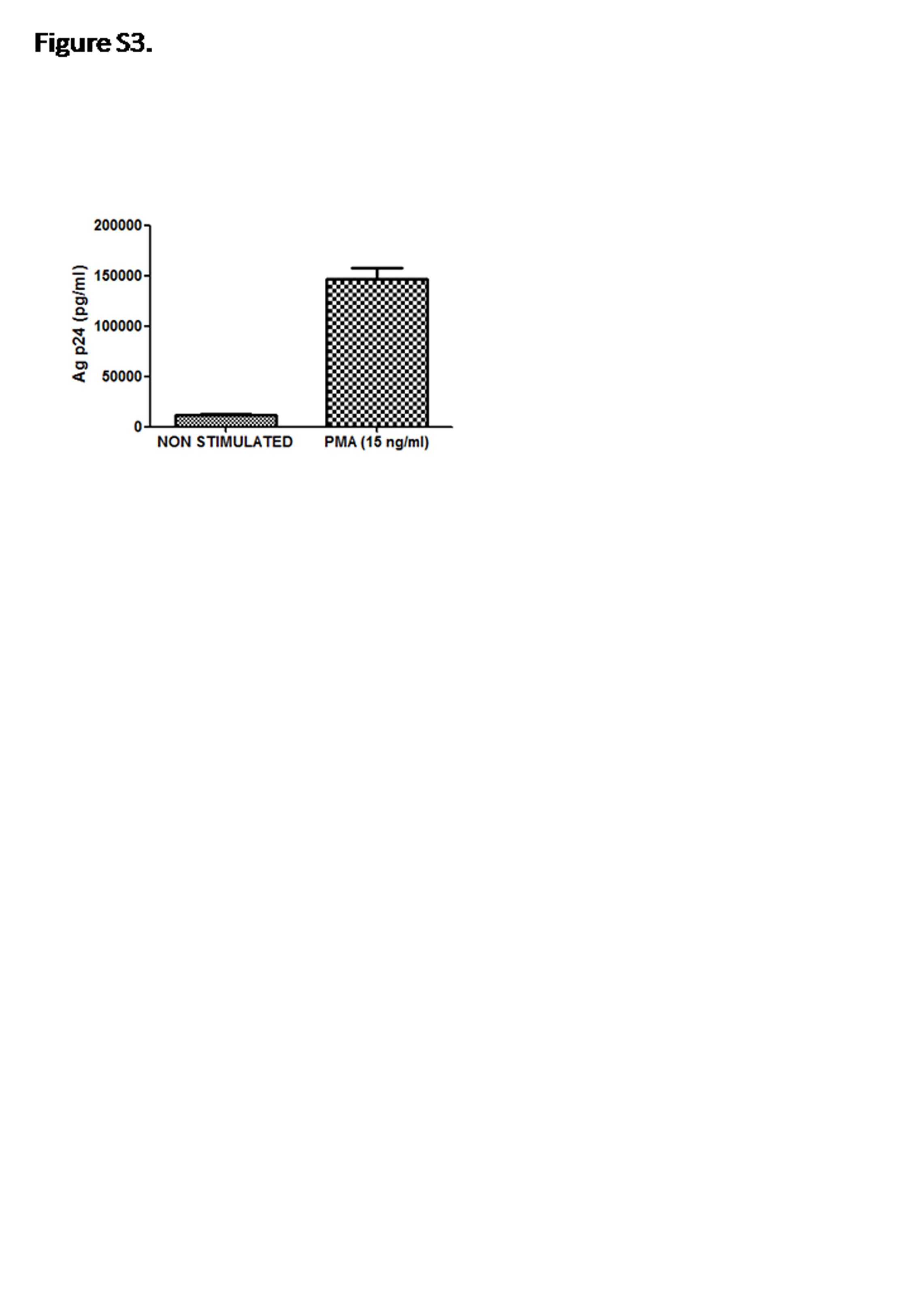

Supplement: Figure S3 — Effect of PMA in LTR-promoter expression. PMA treatment for 3 d increase p24 core protein levels in 8E5 cell line culture supernatants about 10 times. (TIF) [file pone.0085230.s003.tif]

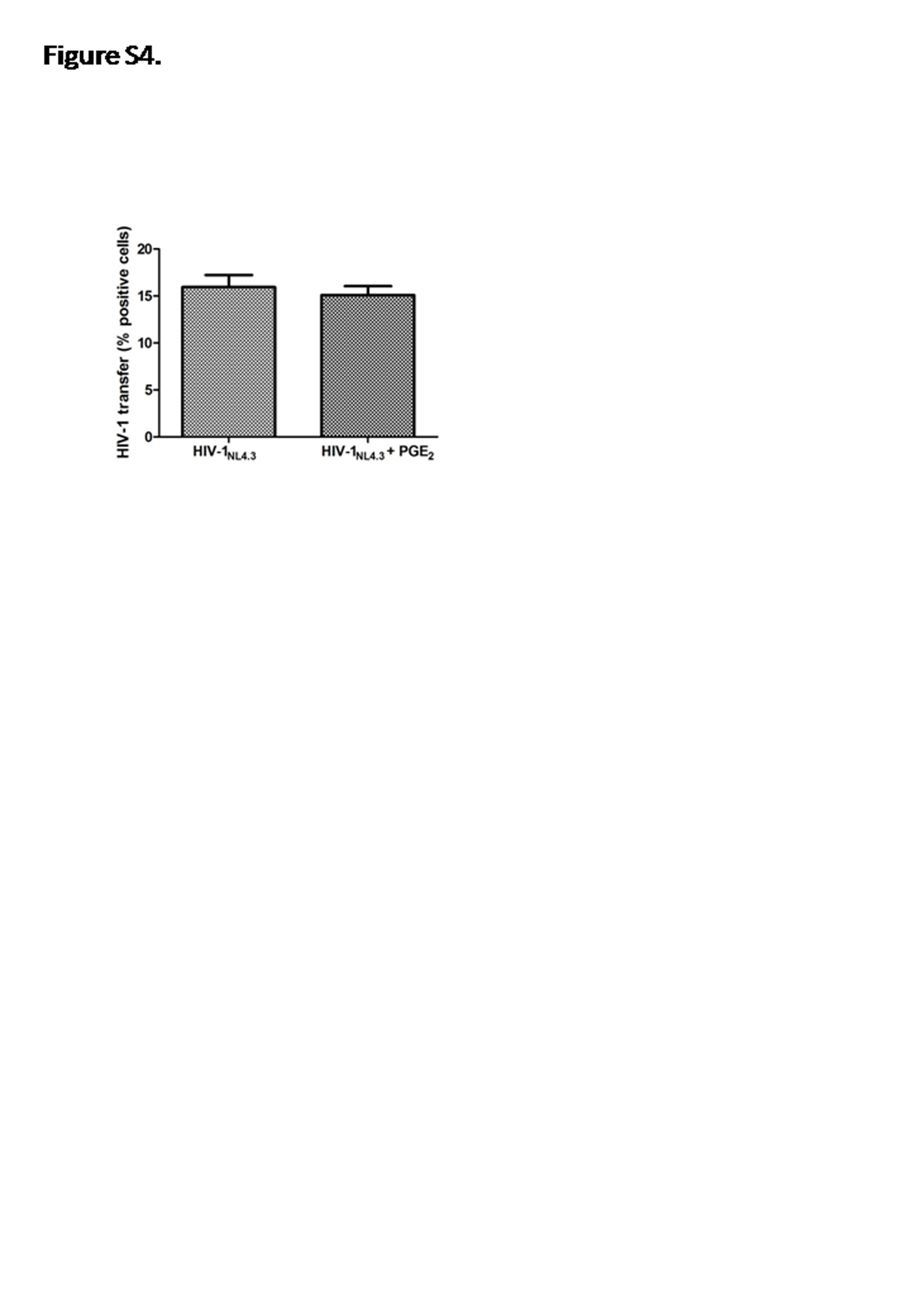

Supplement: Figure S4 — HIV free-viral particle. Purified primary CD4 T cells were infected during 3 d with HIV-1NL4-3 isolate, treated with PGE2, and then co-cultured either with the CMSF labeled target cells. Effector and target cells were seeded at a 2∶1 ratio separated in transwell chambers with a virus-permeable membrane (0.4 µm pore size). Virus transfer was assessed by flow cytometry for intracellular Gag CA p24 in target cells at 6 h after the start of co-culture. Results are shown as a percentage of positive cells for HIV-1 transfer ± SEM of 3 independent experiments. (TIF) [file pone.0085230.s004.tif]

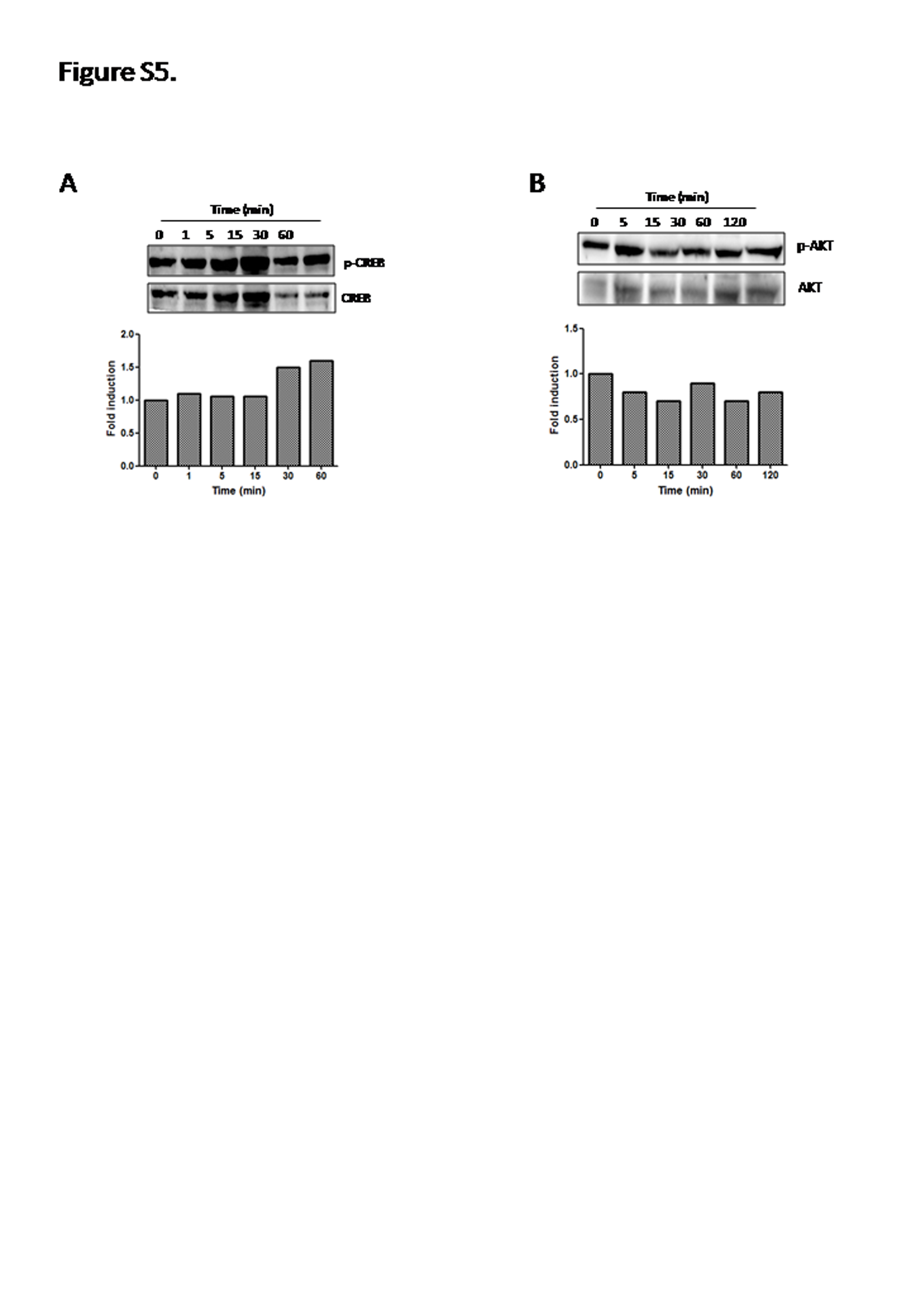

Supplement: Figure S5 — Western blot of A) p-CREB and B) p-AKT in PGE2-stimulated CEM-T cells at the indicated times. Bottom, the graph depicting the results obtained after performing a densitometer analysis of the blots. Western blot representative of three is shown. (TIF) [file pone.0085230.s005.tif]
